# Reservoirs of *Corynebacterium* spp. in the Environment of Dairy Cows

**DOI:** 10.3390/pathogens12010139

**Published:** 2023-01-13

**Authors:** Svenja Woudstra, Anneke Lücken, Nicole Wente, Yanchao Zhang, Stefanie Leimbach, Maya Katrin Gussmann, Carsten Kirkeby, Volker Krömker

**Affiliations:** 1 Department of Veterinary and Animal Sciences, Section for Production, Nutrition and Health, University of Copenhagen, 1870 Frederiksberg C, Denmark; 2 Department of Microbiology, Faculty of Mechanical and Bioprocess Engineering, University of Applied Sciences and Arts, 30453 Hannover, Germany; 3 Department of Veterinary and Animal Sciences, Section for Animal Welfare and Disease Control, University of Copenhagen, 1870 Frederiksberg C, Denmark

**Keywords:** corynebacteria, bovine mastitis, environmental sources of mastitis pathogens, reservoirs of mastitis pathogens

## Abstract

Although *Corynebacterium* spp. can be regularly associated with subclinical and clinical mastitis cases in dairy cows, knowledge on their reservoirs in dairy farms is sparse. Therefore, samples were collected at 10 visits with 14 day intervals from bedding material (n = 50), drinking troughs (n = 20), different walking areas (n = 60), cow brushes (n = 8), fly traps (n = 4), the passage to pasture (n = 9) as well as milking liners (n = 80) and milker gloves (n = 20) in one dairy cow farm. Additionally, quarter foremilk samples from all lactating cows (approximately 200) were collected at each visit. All samples underwent microbiological examination and cultured isolates were identified using MALDI-TOF MS. Most *Corynebacterium* spp. that were cultivated from milk were also isolated from the housing environment and milking-related niches (*C. amycolatum*, *C. confusum*, *C. stationis*, *C. variabile*, *C. xerosis*) or from milking-related niches only (*C. frankenforstense*, *C. pilosum*, *C. suicordis*). *C. bovis* was not cultivated from any environmental niche, while being the dominant species in milk samples. This study demonstrates that many *Corynebacterium* spp. present in milk samples can also be isolated from the cows’ environment. For *C. bovis,* the most relevant *Corynebacterium* species with regard to intramammary infections, it indicates that environmental reservoirs are of little relevance.

## 1. Introduction

Mastitis due to bacterial infections is a common disease in dairy cows that causes considerable economic losses [1]. Pathogens causing mastitis are categorized into major (e.g., *Staphylococcus aureus*, *Streptococcus agalactiae*) and minor pathogens (e.g., non-aureus staphylococci and coryneform bacteria) due to the level of inflammation, measured as somatic cell count in milk, they are associated with [2]. Coryneform bacteria is the designation of a group of genera showing similar phenotypic and biochemical properties including, among others, *Corynebacterium* spp., *Brevibacterium* spp. and *Actinomyces* spp. [3]. *Corynebacterium* (*C*.) species can be regularly isolated from bovine milk [4,5]. The reported quarter-level prevalence within herds ranges from 9 to 24% [6,7]. The species most frequently isolated from dairy cow milk include *C. bovis*, *C. amycolatum*, and *C. xerosis* [8,9]. Their isolation is often associated with moderately increased somatic cell count (SCC). For example, for *C. bovis,* a geometric mean SCC of 78,000 to 174,280 cells/mL milk and for *C. amycolatum* of 50,000 cells/mL milk have been reported [9,10]. *Corynebacterium* spp. can be also cultivated from clinical mastitis cases and at least some species (*C. bovis* and *C. amycolatum*) are present in infected quarters over several months [9,11]. While earlier research had indicated a potential negative effect of intramammary infections with *C. bovis* on milk yield, no effect has been detected in a recent study [10,12].

Corynebacteria have been isolated from the teat canal, the teat cistern, and from the skin of the teat apex [13,14,15]. Although these are indications that the main sources of *Corynebacterium* spp. causing intramammary infections are the skin or mucosal surfaces of dairy cows, it remains unknown if corynebacteria also have reservoirs in the immediate environment of dairy cattle. Knowledge about reservoirs and sources of pathogenic species builds the basis for effective disease prevention and control programs [16]. To the best of our knowledge, no study has yet explored which *Corynebacterium* species can be found in which niches in the environment of dairy cows. Therefore, the aim of the present study was to describe isolation sites of *Corynebacterium* species in the environment of dairy cows in one conventional farm.

## 2. Materials and Methods

An observational study was carried out in one conventional Swedish dairy cow herd with approximately 200 lactating dairy cows. This farm was visited 10 times in 14 day intervals between June and October 2020. The farmer and the advising veterinarian reported before the onset of the study that no particular udder health problems were present. To identify potential environmental reservoirs of bacteria causing intramammary infections, both environmental samples from several locations and milk samples were collected.

### 2.1. Sample Collection

Environmental samples were collected in the housing environment and in or close to the milking parlor at each of the 10 visits.

In the housing environment of the lactating cows, four samples of used bedding material were collected from the rear third of each four adjacent cubicles [17]. The same cubicles were sampled at every visit. Equal parts of bedding material were taken from each of the four cubicles and the material collected in sterile 2 L plastic bags. Then, one drinking trough in each group was sampled using the wet–dry swab technique according to DIN10113-1 as described previously with slight modifications [17]. First, a sterile swab was moistened with Ringer’s solution (half concentrated), rolled over a surface of approximately 20 cm² and then placed in a tube containing sterile Ringer’s solution (half concentrated). Subsequently, a dry sterile swab was rolled over the same surface area and placed in the same tube as the first swab. The touched ends of both swabs were broken off and the tube was closed immediately. Additionally, samples from the walking alley in front of the same drinking troughs were collected with a sterile spoon until at least 11 g of material covering the slatted concrete floor had been placed in sterile plastic bags. In the same manner, soil from the passage to pasture was collected at the passages narrowest point. This sample was not collected at the last sampling, as cows had no longer access to pasture in late autumn. Additionally, used bedding material (straw) was collected from five points in the calving pen at each visit and collected in one 2 L plastic bag with a zip lock until it was full, but still possible to close the bag.

Samples from the walking alley in the waiting and exit area of the milking parlor were collected twice at every visit, once after the milking of Group 1 and again after the milking of Group 2. These samples were collected using the wet–dry swab technique as described above. In addition, samples from milking liners were taken with the wet–dry swab technique. Deviating from the swabbing of a surface of approximately 20 cm^2^, the inner surface of the liners (close to the mouthpiece lip) was sampled by swabbing the liner wall in two rounds with each of both swabs [17]. Liner samples were taken individually from all four liners of one milking cluster after Group 1 and Group 2 had been milked. Additionally, after the milking of each group had been completed, the gloves of the milker were collected and placed in a sterile plastic container.

During the study period, two new cow brushes were installed (one in each group) and we decided to also sample these at the approximate height of the cows’ teats, assuming that material on the brush could splash onto the udder and teats of the cows when rotating. A modified wet–dry swab technique was used. First, the wet swab was held onto the lower part of the brush and the brush brought into automatic rotation by applying pressure with the free hand. The wet swab was held in position until five rotations had passed and subsequently the dry swab sample was collected in the same manner. Both swabs were placed into the same sterile tube containing Ringer’s solution (half concentrated). These samples were collected at the last four samplings. In September, we additionally hung up four commercially available sticky fly traps, left them until the next visit, then collected the traps and placed them in one sterile plastic container each.

At each visit, quarter foremilk samples were collected from all lactating quarters using the aseptic technique. All samples were immediately transported to the laboratory and microbiological examination began within 18 h.

### 2.2. Microbiological Examination of Environmental Samples

The goal of the overall project was to investigate sources and infection dynamics of various mastitis pathogens. Therefore, a broad spectrum of cultivation media was used for the microbiological analysis of environmental samples.

For bedding material, ground samples from the passage to pasture and material collected from the slatted floors in front of the drinking troughs, the following procedure was used: 10 g of sample material was placed into a sterile Stomacher^®^ bag, diluted with 90 mL Ringer’s solution (Merck, Darmstadt, Germany) and mixed for 1 min (easyMIX, AES Chemunex/bioMérieus, Marcy l’Etoile, France) [17]. Decimal dilutions 10^−2^ to 10^−5^ were inoculated on each one plate of Baird Parker Agar (Merck, Darmstadt, Germany), Chromocult^®^ Coliform Agar (Merck, Darmstadt, Germany), and Edwards modified medium (Oxoid, Wesel, Germany) supplemented with colistin sulfate and oxolinic acid (both from Sigma-Aldrich, Munich, Germany) [18].

All tubes containing wet–dry swabs were mixed for 1 min using a Vortex Genie 2 (Scientific Industries, Bohemia, NY, USA). Subsequently, decimal dilutions (10^−1^ to 10^−4^) were prepared using half-concentrated Ringer’s solution, and transferred onto esculin blood agar plates.

Gloves were placed in Stomacher^®^ bags filled with 100 mL half concentrated Ringer’s solution and fixed at the wrist part to the top of the stomacher bag with a bag clip, so only material on the outside surface of the gloves would be dissolved in the solution. After mixing for 1 min, decimal dilutions (10^−2^ to 10^−3^) were prepared and plated onto Chromocult^®^ Coliform Agar, Baird Parker agar, and Edwards modified medium supplemented with colistin sulfate and oxolinic acid. Additionally, dilutions of 10^−3^ to 10^−4^ were plated onto esculin blood agar plates.

From each flytrap, all flies of the same species were picked and transferred into one 2 mL reaction tube containing 1 mL sterile Ringer’s solution. To recover microbes from the outer surface of the flies, the tubes were shaken for 10 s using a Vortex Genie 2 at lowest speed and all liquid was transferred to a new sterile tube for microbiological diagnostics. Subsequently, again 1 mL of Ringer’s solution was added to the tubes containing the flies. The content was then homogenized with glass beads (Hybaid RiboLyser, 10 s Speed 4) to also recover microbes contained inside the flies. The resulting solution was used for the microbiological analysis. Both solutions were individually processed. Decimal dilutions 10^−1^ to 10^−3^ were transferred onto each one plate of Baird Parker Agar, Chromocult^®^ Coliform Agar, and Edwards modified medium supplemented with colistin sulfate and oxolinic acid. Additionally, decimal dilutions 10^−3^ to 10^−5^ were transferred onto esculin blood agar plates.

All esculin blood agar and Baird Parker agar plates were incubated aerobically for 48 h at 37 °C and all Chromocult^®^ Coliform Agar and Edwards modified medium agar plates were incubated aerobically for 24 h at 37 °C.

Per sample, up to 24 different looking colonies were picked from the agar plates. These were transferred into pure cultures through aerobic incubation at 37 °C for 24 h on esculin blood agar plates and, if no growth was present, were reexamined after another 24 h. Subsequently, pure cultures underwent species identification by MALDI-TOF MS.

### 2.3. Microbiological Examination of Milk Samples

The microbiological analysis of milk samples has been described previously [11]. In brief, 10 µL of each milk sample were inoculated on esculin blood agar (Oxoid Deutschland GmbH, Wesel, Germany) and plates were examined after incubation for 24 and 48 h at 37 °C [19]. Morphological and biochemical characteristics were used for preliminary species identification. Consecutively, individual colonies of all isolates were transferred onto a new esculin blood agar plate and incubated for 24 h at 37 °C.

### 2.4. MALDI-TOF MS

One colony of each pure culture from milk and environmental isolates was used for species identification with MALDI-TOF MS (Microflex LT/SH smart, Bruker Daltonik GmbH, Bremen, Germany) using the direct smear method. Spectra were compared against the MBT Compass Library (Revision F, MBT 8468 MSP Library) and a score value of 1.7 was considered a secure species identification [9,20].

As the present study was purely descriptive, no inferential statistical analyses were carried out.

## 3. Results

In total, 251 environmental samples and 8056 milk samples were collected. All isolated *Corynebacterium* species and the sample types from which they were isolated are displayed in Table 1.

From all investigated environmental locations, at least two different *Corynebacterium* species were isolated. The species isolated from the largest number of environmental samples were in descending order: *C. stationis* (n = 65), *C. xerosis* (n = 41), *C. amycolatum* (n = 16) and *C. confusum* (n = 13). For *C. stationis,* the main isolation site was bedding material and the floor in front of the drinking troughs, while *C. amycolatum* and *C. confusum* were mainly isolated from milking liners. *C. xerosis* was frequently isolated from milking liners and milker gloves, but also several times from bedding material, the floors in front of the drinking troughs and from more than half of all samples of the passage to pasture.

The species most frequently isolated from milk samples were *C. bovis* (n = 1410) and *C. amycolatum* (n = 138) [11]. Although *C. bovis* was the dominating species in milk, it was not isolated from any of the environmental samples. Additionally, *C. freneyi* (n = 1), *C. jeikeium* (n = 1), *C. kroppenstedtii* (n = 2), and *C. testudinoris* (n = 6) were isolated from milk, but never from environmental samples.

All *Corynebacterium* species that were not isolated from milk samples during the study period (*C. callunae*, *C. casei*, *C. glutamicum*) were also not isolated from milking liners, but *C. callunae* was once isolated from milker gloves.

## 4. Discussion

Although *Corynebacterium* spp. can be frequently cultivated from milk of dairy cows and some species can be associated with increased somatic cell counts and even clinical mastitis cases, the knowledge on infection pathways of intramammary infections with *Corynebacterium* spp. is sparse. The present study therefore describes the isolation sites of various *Corynebacterium* species in the environment of dairy cows.

Our results show that *Corynebacterium* spp. are ubiquitous in the housing environment of dairy cows. From all investigated locations, at least two different species could be isolated. Furthermore, many of the species cultivated from milk samples were not only frequently isolated from milking liners or milker gloves, but also from the housing environment including, e.g., bedding material, the floor in front of the drinking trough or the passage to pasture.

The species most frequently isolated from the housing environment was *C. stationis*. This bacterium can be isolated from human infections and blood, and has been previously found in milk and teat apex swab samples [21,22]. Additionally, in the present study it has been isolated from milk (n = 3). Not much is known about its pathogenic potential in general [21]. With regard to bovine mastitis, our current knowledge on most *Corynebacterium* spp. remains limited because a secure differentiation of *Corynebacterium* spp. is done only rarely in clinical laboratories and only a few studies exist describing the species distribution of *Coynebacterium* spp. in milk [9].

*C. amycolatum* and *C. xerosis* were occasionally isolated from milk samples and most of their environmental isolates were cultivated from milking liners. While *C. amycolatum* was only rarely isolated from other locations, *C. xerosis* was also regularly found on milkers gloves, in bedding material, on floors and on the passage to pasture. Corynebacteria (e.g., *C. xerosis*) can be regularly found on the teat skin of cows [23]. Thus, the milking liners might have either become contaminated through the contact with teat skin or through the milk that was flushed into the liners during milking. Both could lead to a transmission of bacteria from one teat to another. Kirkeby et al. proposed previously that *Corynebacterium* spp. might be transmitted contagiously through milking liners [6]. Additionally, in another part of the present study, we examined the strain diversity of *C. amycolatum* isolates on a small number of infected quarters (n = 6). Only two different strains were identified, indicating potential contagious transmission [11]. Therefore, the disinfection of liners between cows during milking and post milking teat disinfection might be effective preventive measures against new intramammary infections with some *Corynebacterium* species. Further research is necessary to explore the transmission routes of individual *Corynebacterium* spp. associated with intramammary infections.

On the floor of the waiting area, we only found species that were also isolated in milk samples (*C. stationis* and *C. xerosis*). However, three of the four species isolated from the floor of the exit area of the milking parlor were never isolated from milk. Overall, only a few *Corynebacterium* spp. were isolated from the respective samples, therefore this might only be an incidental finding. On the other hand, many cows drip milk in the waiting area before it is their turn to be milked. While always approximately the same area was sampled and dripped milk has not been expressively collected, residues of milk containing the respective species might have been present on the ground. Whatever was the origin of the *Corynebacterium* spp. on the floor of the waiting area, this location can be a reservoir of *Corynebacterium* spp. found in milk and the teats of cows might get exposed to them (e.g., through splashes) directly before milking.

The overall field study was not set up to specifically identify sources of *Corynebacterium* spp. Therefore, a broad spectrum of cultivation media was used. We used nonselective esculin blood agar plates as well as some media that are mainly used to isolate species of other genera (Baird Parker Agar for the isolation of *Staphylococcus* spp., Edwards modified medium to isolate *Streptococcus* spp. and Chromocult^®^ Coliform Agar for the isolation of coliform bacteria). Still, this is the first study describing environmental reservoirs of corynebacteria at the species level. Although no selective methodology was used, we isolated eleven different species from eleven different environmental sites. The results of this study cannot be used to quantify the importance of different niches as reservoirs of *Corynebacterium* spp., but they shed light on potential environmental reservoirs.

In addition, no strain comparison of *Corynebacterium* spp. isolated from the environment and milk samples was carried out, due to financial limitations. Therefore, it remains unknown if environmental locations harbored the same strains as those isolated from milk. To explore the importance of the identified reservoirs in more depth, future studies should include a strain typing approach to see if environmental reservoirs can be associated with intramammary infections beyond the species level.

With the applied study protocol, we did also not specifically search for *C. bovis*. Still, it is remarkable that *C. bovis*, which was frequently isolated from milk samples, was not found once among the selected isolates from environmental samples including milking liners and milker gloves. This could indicate that even if *C. bovis* is present in the environment of dairy cows it is present in low quantities, or that the environmental reservoir is outside the sampled areas. *C. bovis* grows very slowly on agar plates compared to other microorganisms found in farm environments and the colonies on agar plates are usually small. Therefore, we cannot rule out that it was outgrown by the competitive flora in the samples and therefore not detected. In future research, an approach with a prescreening using PCR and consecutive cultivation under favorable conditions, as recently conducted for *Streptococcus dysgalactiae* [24], could increase the chances of isolating *C. bovis* from environmental samples.

Finally, the present study was only carried out in one herd. Future research on reservoirs of *Corynebacterium* species in the environment of dairy cows should therefore cover a larger number of herds with different production conditions.

## 5. Conclusions

Knowledge about reservoirs and sources of pathogenic species builds the basis for effective disease prevention and control programs. To the best of our knowledge, we report here the first study describing isolation sites of various *Corynebacterium* species in the environment of dairy cows. Several species that cause intramammary infections (e.g., *C. amycolatum* and *C. xerosis*) were isolated from the housing environment (e.g., bedding material and the floor in front of the drinking trough) and milking-related niches (i.e., milker gloves or milking liners). While being the dominant species isolated from milk samples, *C. bovis* was not once cultivated from environmental samples. Although this study provides valuable insight into sources of *Corynebacterium* spp. in dairy cow farms, we suggest that future studies should include molecular comparisons of strains isolated from milk and the environment. For *C. bovis*, the most relevant Corynebacterium species with regard to intramammary infections, the present study indicates that environmental reservoirs are of little relevance.

## Figures and Tables

**Table 1:** *Corynebacterium* spp. isolated from milk and environmental samples.

	Sample Location/Type (n *)											
Species	Bedding Lactating Cows (40)	Bedding Close Up Pen (10)	Drinking Trough (20)	Floor Drinking Trough (20)	Cow Brush (8)	Fly Trap (4)	Passage to Pasture (9)	Waiting Area (20)	Milking Exit (20)	Milking Liner (80)	Milker Gloves (20)	Milk ** (8056)
*C. amycolatum*	1	-	1	1	1	-	1	-	-	11	-	138
*C. bovis*	-	-	-	-	-	-	-	-	-	-	-	1410
*C. callunae*	-	-	-	-	-	1	-	-	2	-	1	-
*C. casei*	1	1	-	-	-	1	-	-	1	-	-	-
*C. confusum*	1	-	1	1	-	-	-	-	-	8	2	2
*C. frankenforstense*	-	-	-	-	-	-	-	-	-	1	-	13
*C. freneyi*	-	-	-	-	-	-	-	-	-	-	-	1
*C. glutamicum*	-	-	-	-	-	-	-	-	1	-	-	-
*C. jeikeium*	-	-	-	-	-	-	-	-	-	-	-	1
*C. kroppenstedtii*	-	-	-	-	-	-	-	-	-	-	-	2
*C. pilosum*	-	-	-	-	-	-	-	-	-	1	-	1
*C. stationis*	29	5	-	13	-	-	4	3	2	3	6	3
*C. suicordis*	-	-	-	-	-	-	-	-	-	1	-	2
*C. testudinoris*	-	-	-	-	-	-	-	-	-	-	-	6
*C. variabile*	-	-	-	-	1	-	-	-	-	1	-	1
*C. xerosis*	5	2	-	5	-	1	5	2	-	15	6	15

* total number of samples taken throughout the project from the specific location/sample type; ** milk sample results taken at the same herd at the same sampling dates have been previously reported in [11] and are here presented again to ease the comparison of results from environmental samples and milk samples for the reader.

## Data Availability

Not applicable.
